# High-Selectivity Nonenzymatic Creatinine Sensor Using Electrografted Ionic Liquid and Nafion for Reliable Clinical Diagnostics

**DOI:** 10.1021/acssensors.5c01503

**Published:** 2025-08-05

**Authors:** Shih-Hao Lin, Jing-Chun Wang, Zong-Hong Lin, Fu-Cheng Kao, Hsiang-Yu Wang

**Affiliations:** a Department of Engineering and System Science, 34881 National Tsing Hua University, 101, Section 2, Kuang-Fu Road, Hsinchu 300044, Taiwan; b Institute of Nuclear Engineering, 34881 National Tsing Hua University, 101, Section 2, Kuang-Fu Road, Hsinchu 300044, Taiwan; c Department of Biomedical Engineering, 33561 National Taiwan University, No.1, Section 4, Roosevelt Road, Taipei 106319, Taiwan; d Department of Orthopaedic Surgery, 38014 Chang Gung Memorial Hospital, No. 5, Fuxing St., Guishan Dist., Taoyuan City 33305, Taiwan; e College of Medicine, Chang Gung University, No.259, Wenhua first Rd., Guishan Dist., Taoyuan City 33302, Taiwan

**Keywords:** creatinine, styrenyl-triphenylphosphonium-based ionic liquid, Nafion, electrografting, CuONPs

## Abstract

The rapid and accurate detection of creatinine is essential for monitoring kidney function and diagnosing renal impairments. Nonenzymatic catalysts have been used to improve sensitivity and reduce environmental susceptibility in creatinine detection. However, limited selectivity hinders their broader application in practical scenarios. This study introduced a novel nonenzymatic creatinine sensor that utilized copper­(II) oxide as a catalyst, incorporating styrenyl-triphenylphosphonium-based ionic liquid (STPP-IL) and Nafion on the electrode surface to enhance selectivity and minimize interference. Electrografting technique ensured uniform STPP-IL coverage on the electrode, as confirmed by scanning electron microscopy (SEM) and energy-dispersive X-ray spectroscopy (EDS). The modified creatinine sensors demonstrated a detection range from 1.5 to 800 μM with low standard error and high sensitivities in the presence of interferents. Notably, creatinine levels in the artificial and natural human sweat were selectively quantified using the proposed sensor, supporting its potential for clinical applications. The sensors retained over 90% of their initial current response after 40 days of storage. Additionally, reproducibility experiments demonstrated a relative error of 1.3% across five independent sensors. These results show the sensor’s enhanced selectivity, stability, and reproducibility, making it a promising alternative for reliable creatinine detection in point-of-care tests.

## Graphical abstract

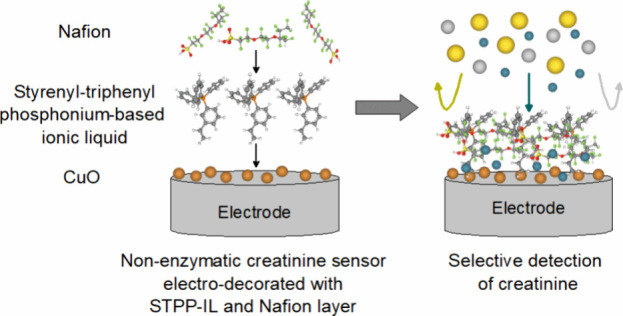

Creatinine, a metabolic byproduct of creatine, reflects the dynamic equilibrium between renal clearance and tissue production.^1^ The human body generates a consistent daily creatinine output, primarily excreted through the kidneys, with minimal tubular reabsorption. Normal serum creatinine levels, indicative of renal clearance efficiency, typically range from 45 to 110 μM.^2–4^ Elevations above 150 μM suggest compromised kidney function, while below 40 μM may indicate low muscle mass or potential myopathies. In severe renal injury, serum creatinine can exceed 500 μM, necessitating urgent dialysis or transplantation.^5^ Accurate and timely creatinine detection is vital for early diagnosis and management of renal impairment.^6,7^ Conventional creatinine assays involve colorimetry and enzymatic sensors.^8^ While enzymatic catalysts possess excellent selectivity, they can be limited by manufacturing complexity, environmental susceptibility, and strict storage requirements.^9,10^

Nonenzymatic creatinine sensors have demonstrated higher sensitivity and reduced environmental susceptibility, particularly when utilizing copper-based catalysts, which are promising due to copper’s strong ligand coordination properties.^11^ However, nonenzymatic catalysts often suffer from poor specificity, leading to potential false positives caused by biomolecule interference in body fluids.^12^ To address the drawback, molecularly imprinted polymers (MIPs) have emerged as a viable approach for enhancing selectivity. MIPs are synthesized by imprinting the target molecule within a polymer matrix, creating highly specific binding sites.^13^ Integrating nanomaterials with MIP-based sensors has significantly improved performance, achieving nanomolar detections with minimal interference.^14^ Despite these advancements, MIP development remains time-consuming and requires optimization of numerous fabrication parameters.^15^ Besides, the detection process involves multiple steps, which can be cumbersome. Surface modification using cationic/anionic polymers and aptamers has also been investigated to improve selectivity, but these methods often need to be revised in terms of cost-effectiveness, selectivity, speed, or ease of fabrication.^16,17^ Therefore, nonenzymatic sensors continue to face limitations in achieving widespread clinical adoption.

Ionic liquids (ILs), noted for their customizable properties, have gained prominence in electrochemical sensor design due to their ability to facilitate electron transfer and form strong interactions. Additionally, their application in the analytical extraction of diverse compounds, including biomarkers, further highlights their potential.^18,19^ However, the application of ILs in biosensing necessitates further refinement to effectively integrate selective extraction with conductivity maintenance.^20^ This limitation can be attributed to the need for interdisciplinary research in electrochemistry and analytical chemistry to address challenges such as appropriate affinity for target analytes, poor electrode adhesion, and high consumption rates. Our recent study has demonstrated the effectiveness of the electrografting technique for immobilizing ILs in developing a nonenzymatic sensor for lactate detection.^21^ This approach offers advantages such as low IL consumption and reproducible sensors, highlighting its potential for developing highly selective and reliable nonenzymatic sensors. However, the impact of temperature on sensor performance and storage stability remains unexplored. Assessing these factors under different conditions is crucial for ensuring their suitability for clinical diagnostics and point-of-care testing.

This study presents a novel approach to enhance the selectivity of nonenzymatic creatinine sensors by electrodecorating IL and Nafion. The screen-printed electrode decorated with rGO and copper­(II) oxide nanoparticles (CuONPs) provides a suitable detection range for health monitoring. The electro-decorated styrenyl-triphenylphosphonium-based ionic liquid (STPP-IL) and Nafion provided detection selectivity, effectively preventing interactions between interfering biomolecules and CuONPs. Cyclic voltammetry validated the selectivity of the creatinine sensor. Calibration curves, exhibiting linearity over the range from 1.5 to 800 μM, were established in the presence of physiological concentrations of interferents. To simulate practical applications, the recovery rates of the sensors were evaluated in artificial sweat samples. Moreover, natural sweat samples were collected and analyzed to further validate the sensor’s applicability in real-world conditions. Finally, the sensor performance and storage stability were assessed at various temperatures. All sensors retained over 90% of their initial current response after 40 days of storage.

## Experimental Section

### Materials

Calcium carbonate (CaCO_3_), single-layer graphene oxide (SL-GO), l-ascorbic acid (L-AA), copper nitrate (Cu­(NO3)­2), pectin, 4-chloromethylstyrene, triphenylphosphine, Nafion perfluorinated resin, phosphate-buffered saline (PBS), creatinine, glucose, uric acid (UA), urea, and L-lactic acid (L-lac) were all purchased from Sigma, USA.

### Fabrication of Screen-Printed Electrodes Decorated with rGO and CuO Nanoparticles

Porous screen-printed electrodes were fabricated following our previously described methodology.^22^ A graphene suspension was prepared by dispersing 50 mg of SL-GO and 50 mg of L-AA in 50 mL of DI water, which was left overnight for reduction. The resulting mixture was then combined with a 0.15 M NaCl solution in a 3:4 ratio to produce the final solution for electrode modification. The screen-printed electrodes were immersed in graphene suspension and subjected to 15 segments of cyclic potential from −1.0 to −1.6 V at 10 mV/s for further improvement.

The copper plating solution contained 107 mM Cu­(NO_3_)_2_, 0.1 M H_2_SO_4_, and 1 mg/mL pectin. CuNPs were formed by applying 300 cycles of constant potential at −0.6 V (vs SCE) with a total of 3.3 mC. Subsequently, these CuNPs were transformed into CuONPs through 50 segments of cyclic potential from 0 to −0.5 V (vs SCE) at 50 mV/s in a 1 M NaOH solution. The applied potential and pH solution were referred to in previous literature.^23,24^

### Synthesis of Ionic Liquids

Styrenyl-triphenylphosphonium chloride ([STPP]­Cl) was synthesized under a nitrogen atmosphere by reacting equimolar amounts of 4-chloromethylstyrene and triphenylphosphine (7.128 mM) in 10 mL of *n*-hexane. The reaction mixture was stirred overnight at 60 °C. The synthesized [STPP]Cl was then extracted from the *n*-hexane solution into deionized water and subsequently isolated by evaporating the water at 120 °C under a nitrogen atmosphere.

### Electrodecoration of STPP-IL and Nafion

A solution containing 50 mg of [STPP]Cl was prepared in 2 mL of dimethyl sulfoxide (DMSO) for electrografting. Linear sweep voltammetry was performed from −1 to −3 V at a scan rate of 50 mV/s to determine the optimal electrografting potential, which was found to be −1.78 V. The amount of electrografted IL was optimized by varying the electrografting duration from 2 to 8 s at −1.78 V (vs Ag/AgCl). [STPP]­Cl-modified creatinine sensors effectively reduced interference from common species, except uric acid. Therefore, Nafion was incorporated to suppress uric acid interference. The sensors were immersed in a 1 wt % Nafion perfluorinated resin solution in ethanol and subjected to a constant potential of 1.5 V for different durations from 10 to 25 s to deposit a Nafion layer. The sensors were subsequently air-dried at room temperature. Based on the performance of the modified sensors evaluated by cyclic voltammetry, the optimal fabrication durations were 6 s for electro-decoration of STPP-IL and 20 s for Nafion.

### Sensor Sensitivity, Selectivity, Recovery Rate Assessments, and Natural Sweat Test

Electrochemical examinations in this study were conducted using an Electrochemical Workstation (CHI 6081E, CH Instrument Inc., USA) in a conventional three-electrode setup: an Ag/AgCl reference electrode (ALS Co., Ltd., JAPAN), a carbon counter electrode, and a carbon electrode modified with rGO and CuONPs, with or without electrodecorated [STPP]Cl and Nafion as the working electrode. The working electrode surface area was 4 mm^2^. Cyclic voltammetry (CV) was performed at a scan rate of 50 mV/s over a potential range of −0.6 to 0.6 V. The peak current associated with Cu oxidation near 0.1 V was used to quantify creatinine concentration. Calibration curves were constructed using 10 mM PBS containing varying creatinine concentrations from 0 to 800 μM. To evaluate selectivity, modified sensors with [STPP]­Cl, Nafion, or both were used to quantify 10 μM creatinine at the presence of interferents, including 0.09 mM glucose, 50 μM uric acid, 22.2 mM urea, 5 mM lactic acid, and 10 μM ascorbic acid.

The sensor’s recovery rates were determined by comparing the measured creatinine concentrations, ranging from 9.4 to 18 μM in artificial sweat samples.

Natural sweat samples were collected and analyzed to validate the practical applicability of the proposed creatinine sensor. A calibration curve was constructed using the standard addition method by adding the sweat samples with creatinine concentrations ranging from 10 to 50 μM, without additional sample preparation or purification. Human subject testing was conducted in accordance with ethical regulations and protocols approved by the Institutional Review Board of Chang Gung Memorial Hospital (IRB No. 202302261B0A3). Participants were recruited following IRB guidelines, and passive sweat rate was determined gravimetrically using the cotton disk.

### Evaluation Sensor Performance and Storage Stability at Various Temperature

To evaluate the effects of temperature on sensor performance, calibration curves were constructed at different temperatures (4, 30, and 50 °C). For storage stability, sensors stored at 4, 30, and 50 °C were used to quantify 10 μM creatinine solutions over 45 days. The peak current on the first day was normalized to 100%, and subsequent measurements tracked the time required for the current to drop to 90% of its initial value. Five sensors were tested to assess reproducibility.

## Results and Discussion

### Nonenzymatic Creatinine Sensing with CuONP Catalysts

CuONPs were utilized as a nonenzymatic catalyst for quantitatively detecting creatinine in a 10 mM PBS solution. The presence of CuONPs was confirmed by scanning electron microscopy (SEM) and identified through energy-dispersive X-ray spectroscopy (EDS) (Figure 1A,B). Cyclic voltammetry (CV) revealed a reversible Cu/Cu^2+^ redox pair, with the peak current showing a concentration-dependent response to creatinine from 1 to 600 μM (Figure 1C). Creatinine enhanced the response current without shifting the potential, indicating that additional metallic Cu was exposed on the electrode surface. This phenomenon was attributed to the formation of a soluble copper-creatinine complex, revealing the underlying Cu layer for further redox reaction.^25^ The following reactions govern the oxidation process:

$${\mathrm{Cu}}^{2+}+2\mathrm{creatinine}+2H_{2}O\to [\mathrm{Cu}(\mathrm{creatinine}{)}_{2}(H_{2}O{)}_{2}{]}^{2+}$$ 1

$$\mathrm{Cu}↔{\mathrm{Cu}}^{2+}+2e^{-}$$ 2

**Figure 1:**
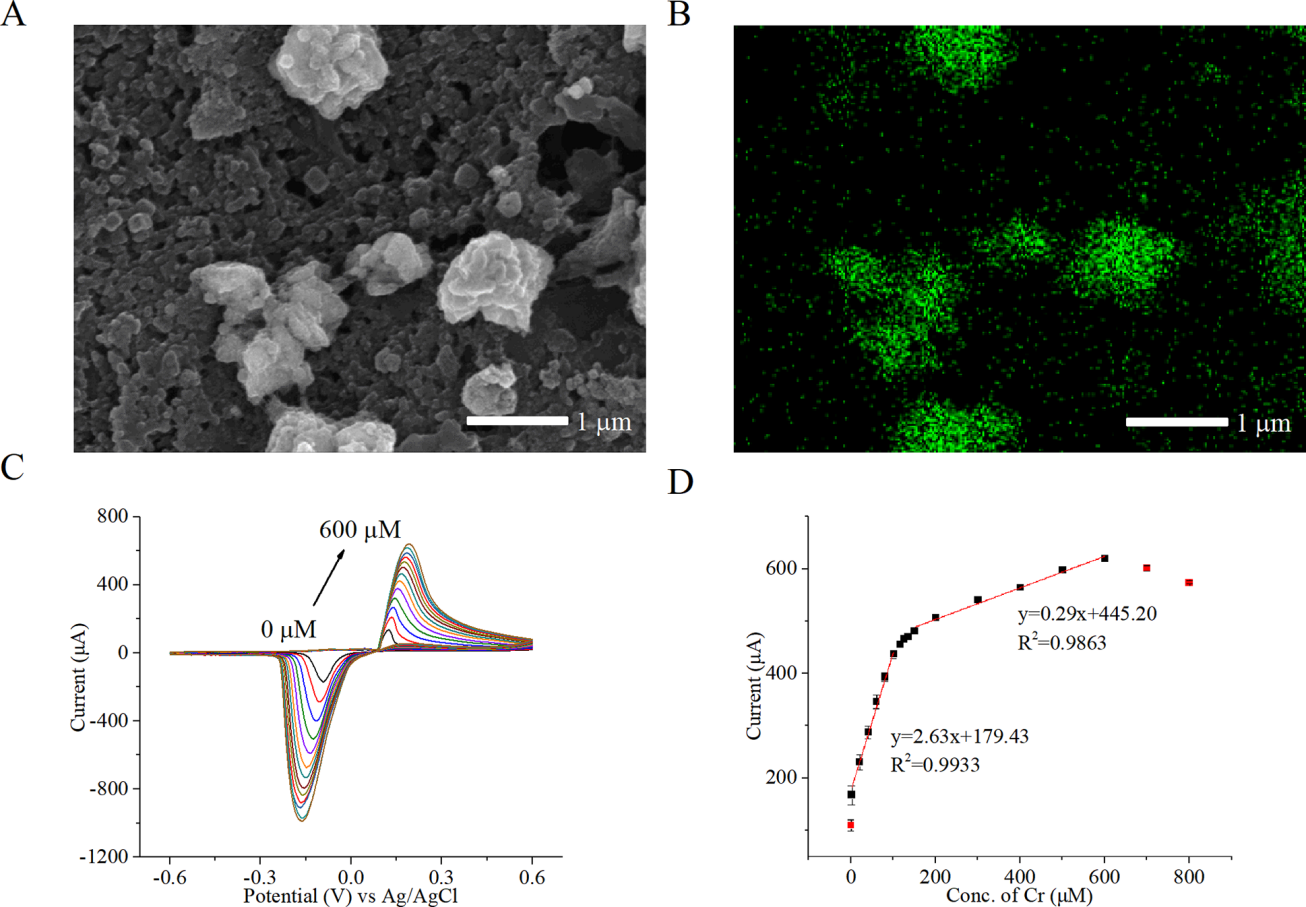
(A) SEM image and (B) EDS mapping of the electrode surface following copper­(II) oxide nanoparticles (CuONPs) electrodeposition. Green spots in the EDS map indicates the Cu element. (C) Cyclic voltammetry analysis of the Cu/Cu^2+^ redox couple in varying creatinine concentrations. (D) Calibration curve illustrating two linear response regions for creatinine detection: one from 1.5 to 100 μM and the other from 150 to 600 μM. The data were obtained from three sensors with triplicate measurements. Error bars were calculated from the overall nine measurements.

After confirming the electrochemical sensor’s ability to detect creatinine, a calibration curve was established over a detection range from 1 to 600 μM, demonstrating the method’s suitability for creatinine diagnosis. The calibration curve demonstrated a linear relationship, exhibiting a higher sensitivity of 2.63 μA/μM in the physiological concentration range and a stable sensitivity of 0.29 μA/μM at elevated concentrations (Figure 1D). The shift suggests that the rate-determining step transitioned from complex formation to desorption of the copper-creatinine complex.^26^ Nevertheless, as a nonenzymatic catalyst, CuONPs offered good detection range and sensitivity for creatinine diagnosis.

### STPP-IL Characterization

Previous studies have demonstrated that quaternary triphenylphosphonium-based ILs can extract organic substances and promote phase separation effectively; however, the challenges, including instability, leakage, and high IL consumption, remain to be addressed.^27–30^ This study employed a novel approach to immobilize the STPP-IL on the electrode surface to address the above-mentioned challenges. By substituting phenyl groups with styrenyl groups, covalent bonding of IL to electrode was achieved. The molecular structure of STPP-IL was confirmed via ^13^C NMR analysis (Figure S1). After the successful synthesis of STPP-IL, linear sweep voltammetry was employed to determine the feasibility of electrografting. Typically, monomers require a conducting salt to polarize the substrate for polymerization in organic solvents. In contrast, ILs can be electrografted without additives due to their inherent conductivity. When applying a dynamic potential from −1 to −3 V, the cathodic current indicated that electrografting began at approximately −1.5 V, with a gradual increase and a mild peak at −1.78 V, which was selected as the optimal electrografting potential (Figure 2A). SEM image shows that the surface morphology of the electrode did not significantly change after STPP-IL electrografting (Figure 2B). EDS analysis was conducted to confirm the successful electrografting of STPP-IL onto the electrode surface. A significant and even increase in the Cl^–^ signal indicated the uniform distribution of the ionic liquid on the electrode (Figure 2C). Cyclic voltammetry for creatinine detection at concentrations ranging from 1 to 600 μM confirmed that the sensor modified with STPP-IL retained its detection capabilities (Figure 2D). The response current indicates that creatinine molecules can penetrate the STPP-IL layer and interact with the copper­(II) oxide catalyst. The sensor’s catalytic activity was preserved despite incorporating the organic moiety of IL. Furthermore, introducing covalent bonds and the electrografting process enabled durable electrodes and minimized the consumption of ionic liquids.

**Figure 2:**
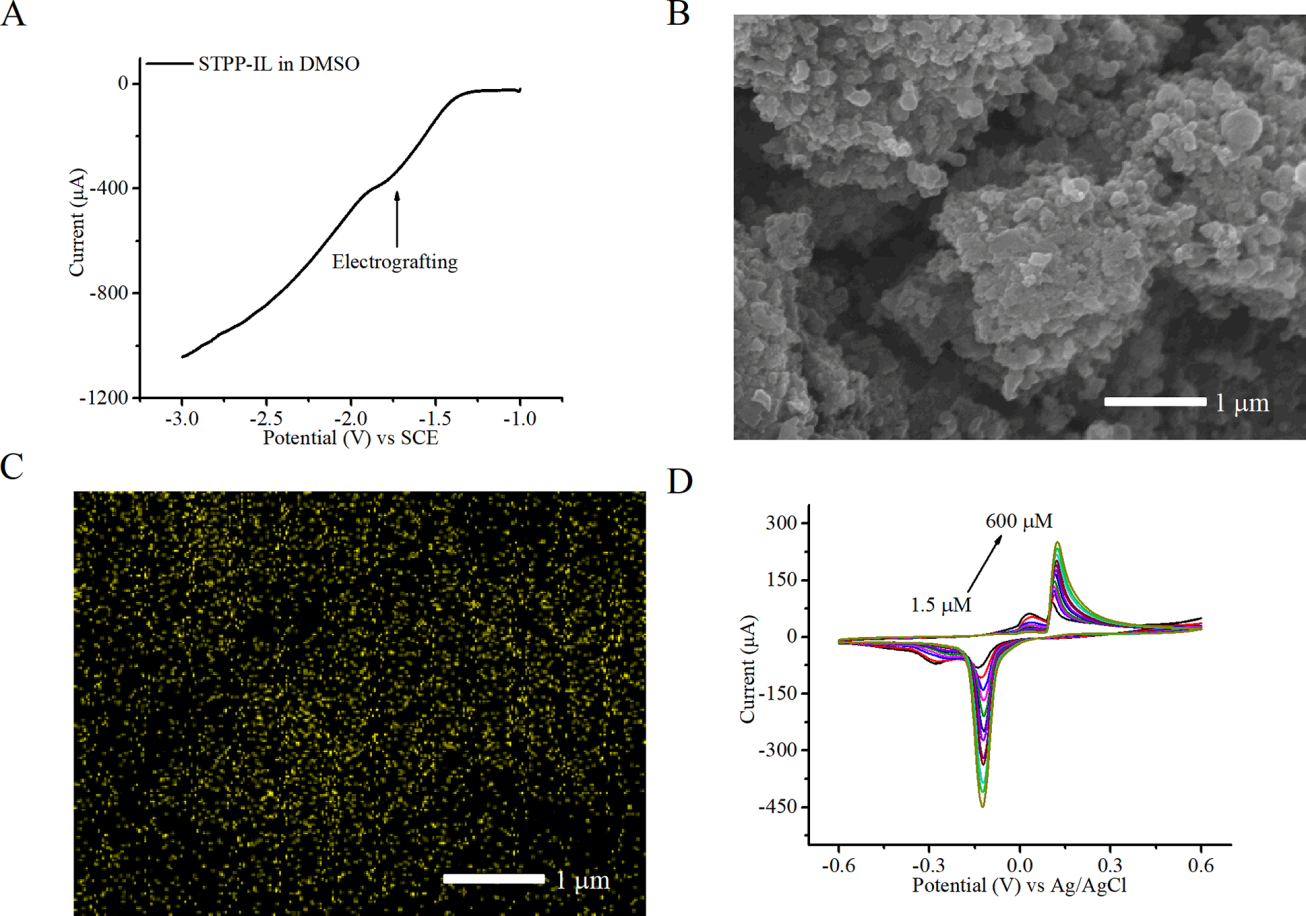
(A) Electrografting of STPP-IL by linear sweep voltammetry between −1 and −3 V. (B) SEM images and (C) EDS analysis displaying Cl^–^ in yellow spots after STPP-IL electrografting. (D) Cyclic voltammetry shows the increasing current response with creatinine concentration by the CuONP sensor electrografted with STPP-IL.

### Selectivity of IL/Nafion-Modified Creatinine Sensor

To evaluate the selectivity of the creatinine sensor modified with STPP-IL under different electrografting durations (0–8 s), cyclic voltammetry was conducted to quantify 10 μM creatinine (Cr) in the presence of common interferents, including 0.09 mM glucose (Glu), 50 μM uric acid (UA), 22.2 mM urea, 5 mM lactic acid (LA), and 10 μM ascorbic acid (AA). The oxidation peak current at approximately 0.1 V was monitored to assess sensor performance. As shown in Figure 3A, the current decreased with longer electrografting time, reflecting increased STPP-IL immobilization. Unlike physically adsorbed ILs that may leach over time, covalently bound STPP-IL ensures long-term stability.^31^ However, excessive IL loading can hinder ion dissociation due to strong electrostatic interactions, reducing charge carrier availability for redox reactions.^32^ The sensor modified with STPP-IL for 6 s maintained relative errors below 5% for all interferents, except for uric acid, which exhibited a relative error of 18.11%. The unique properties of ionic liquids, such as their charged nature and ability to generate localized electric fields, may contribute to their selective binding with certain molecules. However, the exact mechanism underlying this selective affinity remains unclear and warrants further investigation. Experimental results suggest that STPP-IL exhibits a specific affinity for uric acid and creatinine, likely due to the presence of cyclic amide functional groups, which differentiate these compounds from other interferents.

**Figure 3:**
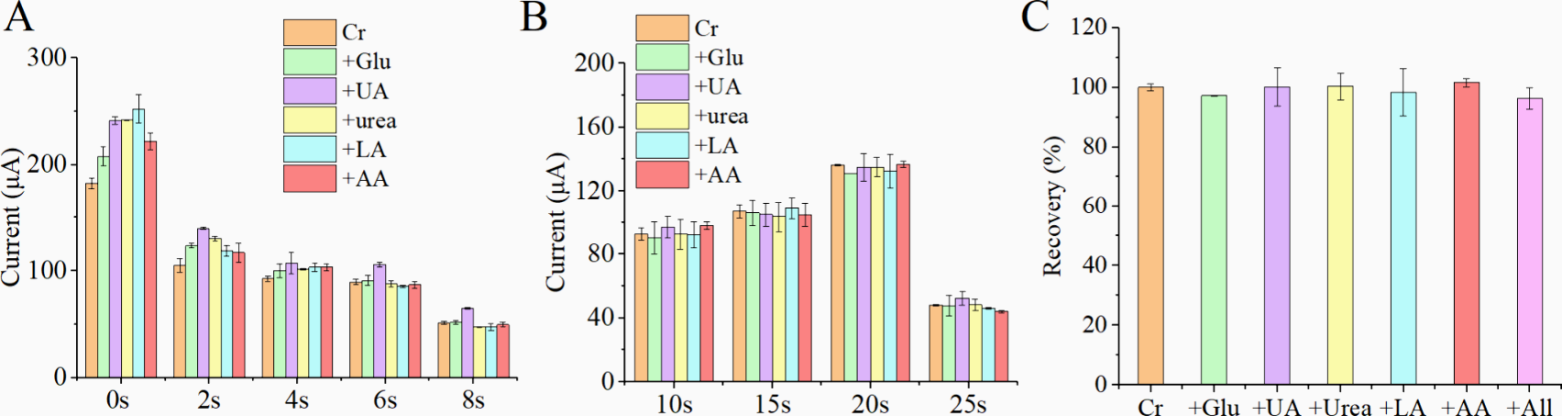
Oxidation peak current measured during cyclic voltammetry for quantifying 10 μM creatinine (Cr) in the presence of various interferents including 0.09 mM glucose (Glu), 50 μM uric acid (UA), 22.2 mM urea, 5 mM lactic acid (LA), and 10 μM ascorbic acid (AA) using creatinine sensors modified with (A) styrenyl-triphenylphosphonium ionic liquid (STPP-IL) at different electrografting durations (0–8 s) and (B) Nafion at varying electrodeposition durations (10–25 s). (C) Recovery rates of sensors modified with electrografted STPP-IL for 6 s and electrodeposited Nafion for 20 s, detecting 10 μM creatinine in the presence of the interferents. The data were obtained from three sensors with triplicate measurements. Error bars were calculated from the overall nine measurements.

The creatinine sensors electrografted with STPP-IL effectively mitigated common interference, except for uric acid. To address this, Nafion was introduced to eliminate uric acid’s influence on sensor performance. The sulfonic groups in Nafion create a negatively charged film that repels anions, preventing uric acid from interacting with the nonenzymatic catalyst.^33^ Additionally, electrodeposition was employed to precisely control the Nafion layer thickness. As shown in Figure 3B, applying a Nafion layer significantly reduced uric acid interference while preserving the sensor’s creatinine detection capability. This finding aligns with previous studies indicating that creatinine, being positively charged at neutral pH, can still permeate the Nafion layer.^34^ Electrodeposition durations of 10 to 20 s led to an increased current response, with 20 s identified as the optimal duration. This condition produced the highest peak current while effectively minimizing uric acid interference to just 0.25%, making it the selected parameter for subsequent experiments.

Although Nafion effectively minimized uric acid interference, negatively charged species such as lactic acid and urea were still able to penetrate the layer, impacting detection accuracy.^35,36^ Our results suggest that integrating STPP-IL and Nafion effectively blocks both positively and negatively charged interferents. To validate selectivity, sensors modified with STPP-IL and Nafion were tested against individual interferents. The relative error remained below 5% for samples containing individual interferents, while samples with all interferents showed an error of only 3.69% (Figure 3C). Additionally, Figure S2 presents calibration curves comparing the undecorated CuO electrode, the CuO electrode decorated with STPP-IL, and the CuO electrode decorated with both STPP-IL and Nafion, illustrating the effects of each modification. Sensors modified with either ionic liquids or Nafion alone exhibited inherent limitations—ionic liquids reduced sensitivity, while Nafion provided insufficient selectivity. In contrast, the combined modification of STPP-IL and Nafion significantly enhanced both selectivity and current response. These findings highlight the synergistic benefits of this approach, effectively addressing interference and sensitivity challenges. This modification strategy enables reliable creatinine detection with multiple interferents, achieving one-step detection without requiring pretreatment.

### Effect of Interference on Sensor Performance

After optimizing the sensor decoration process, calibration curves were constructed in the presence and absence of interferents (Figure 4A,B). The modified sensor exhibited a broad detection range with two linear regions: 1.5–100 μM and 150–800 μM. The sensor demonstrated a dynamic response to creatinine concentrations, with a sensitivity of 0.09 μM/μA in the higher concentration range, maintaining strong linearity both with and without interferents (R^2^ = 0.9961 and 0.9945). The two distinct linear sensitivity regions arise from a shift in the rate-determining step during the sensing process. At low concentrations, creatinine forms a soluble copper–creatinine complex that exposes the underlying metallic Cu, enhancing its redox activity. As the concentration increases, the surface becomes saturated, and the rate-limiting step shifts to complex desorption or diffusion.

**Figure 4:**
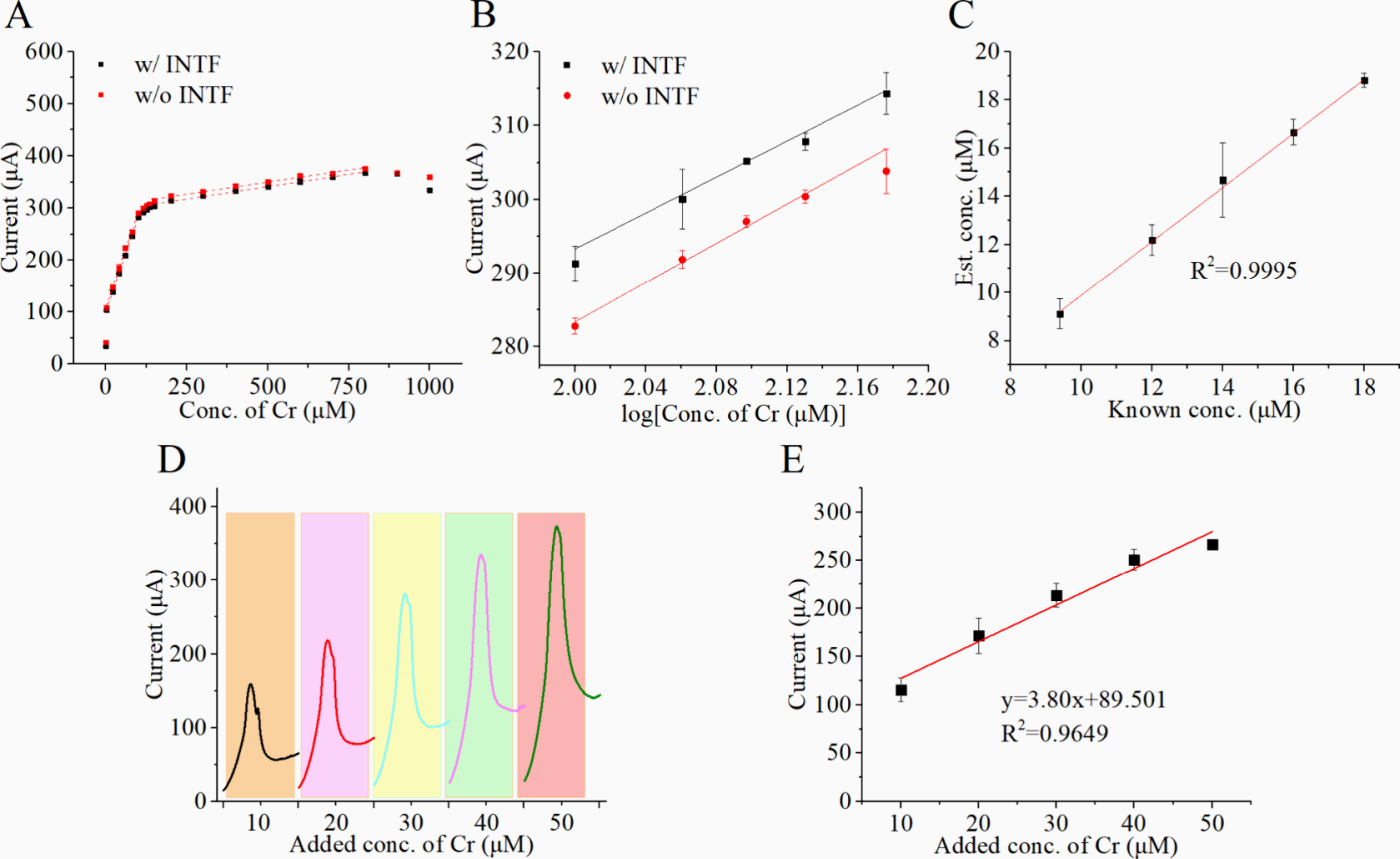
(A) Calibration curve of creatinine sensors modified with electrodecorated STPP-IL and Nafion, measured in the presence and absence of interferents (INTF). (B) Enlarged view of the calibration curve and logarithmic transformation of concentration data from (A) in the range of 100–150 μM. (C) Comparison of estimated and known creatinine concentrations in solutions containing physiological levels of interferents, with a linear regression line illustrating the correlation. (D) The region from −0.1 to 0.4 V, corresponding to the oxidation peak, was extracted from cyclic voltammetry to show the increasing response current in sweat samples by adding 10 to 50 μM creatinine. (E) Standard addition calibration curve in real human sweat by adding 10–50 μM creatinine. The data were obtained from three sensors with triplicate measurements. Error bars were calculated from the overall nine measurements.

In the lower concentration range, sensitivities of 1.80 and 1.81 μM/μA were achieved with and without interferents, respectively, both exhibiting strong linear correlations (R^2^ = 0.9998 and 0.9982). For the transitional region between the two linear ranges, a logarithmic transformation of the concentration data yielded sensitivities of 121.9 and 133.21 log­(μM)/μA, with corresponding R^2^ values of 0.96 and 0.99 in the absence and presence of interferents.

The minimal variation observed between calibration curves with and without interferents highlights the sensor’s exceptional selectivity and robustness against common interferents. Among reviewed creatinine sensors, only one using molecularly imprinted polymers demonstrated the capability to quantify creatinine directly without pretreatment (Table S1). In contrast, the sensor presented in this study achieved significantly enhanced sensitivity, showing improvements of 70.4-fold and 3.5-fold in specific concentration ranges.

Sweat is a promising body fluid for noninvasive creatinine monitoring and may serve as an alternative to blood sampling. Normal sweat creatinine levels typically range from 9.4 to 18 μM. Artificial sweat samples containing known creatinine concentrations (9.4, 12, 14, 16, and 18 μM) and common interferents were tested to evaluate the sensor’s performance further. The sensor accurately predicted creatinine levels, with recovery rates ranging from 97% to 105% (Table S2). A strong linear correlation between the known and estimated concentrations is shown in Figure 4C, with a slope of 1.1277 and an R^2^ value of 0.9995.

To further assess the sensor’s performance for practical application, creatinine levels in natural sweat were quantified using the standard addition method with added concentrations ranging from 10 to 50 μM (Figure 4D,E). By extrapolation, the endogenous creatinine concentration was determined to be 23.51 μM. Compared to using PBS as a media, the sensor demonstrated a nearly 2-fold increase in sensitivity when tested in natural sweat, reaching 3.8 μA/μM. Conductivity measurements showed a marked difference between the two media, with 10 mM PBS at 3.1 mS/cm and sweat at 17.2 mS/cm, suggesting that the higher ionic strength of sweat contributed to the enhanced electrochemical response. At higher added creatinine concentrations, the calibration curve exhibited a diminished slope. This phenomenon was similar to the trend observed in PBS and is likely attributed to the desorption of copper–creatinine complexes from the electrode surface. Notably, such signal attenuation was only observed when the total creatinine concentration exceeded 60 μM, a level associated with potential health risks.^37,38^ This observation reinforces the sensor’s suitability for detecting physiologically relevant creatinine levels in healthy individuals. The sensor maintained reliable performance within this range and demonstrated consistent accuracy even in common interferents, confirming its robustness under real-world conditions. Notably, the sweat sample in this study was collected from a single healthy volunteer. While interindividual differences in electrolyte composition and sweat rate may slightly affect quantification, they have minimal impact on overall sensor performance and can be addressed through calibration or normalization. Moreover, the correlation between sweat and plasma creatinine remains under investigation, as the composition of sweat may reflect glandular activity rather than plasma levels.^39^ Nevertheless, proposed sensors offer strong potential for early screening and noninvasive monitoring of kidney function. Overall, the modified creatinine sensor presents a promising solution for practical, noninvasive applications, offering a broad detection range, high selectivity, and the advantage of requiring no complex sample preparation.

### Stability, Durability, and Reproducibility of Creatinine Sensors under Varying Temperatures

Conventional enzymatic catalysts often suffer from environmental susceptibility and limited lifespans, requiring specific storage conditions. To evaluate the superior reliability of the nonenzymatic creatinine sensor modified with STPP-IL and Nafion, sensor performance was tested at 4, 30, and 50 °C (Figure 5A). The calibration curve exhibited two distinct linear response regions: 1.5–100 μM and 150–800 μM. The sensor’s sensitivity remained stable across different storage temperatures. In the lower concentration range, sensitivity values were 1.82 μA/μM at 4 °C, 2.07 μA/μM at 30 °C, and 1.89 μA/μM at 50 °C. In the higher concentration range, sensitivities were 0.09 μA/μM at both 4 and 30 °C, and 0.10 μA/μM at 50 °C. These results demonstrate that sensor sensitivity was minimally affected by temperature fluctuations, highlighting its robustness and suitability for practical applications under varying environmental conditions.

**Figure 5:**
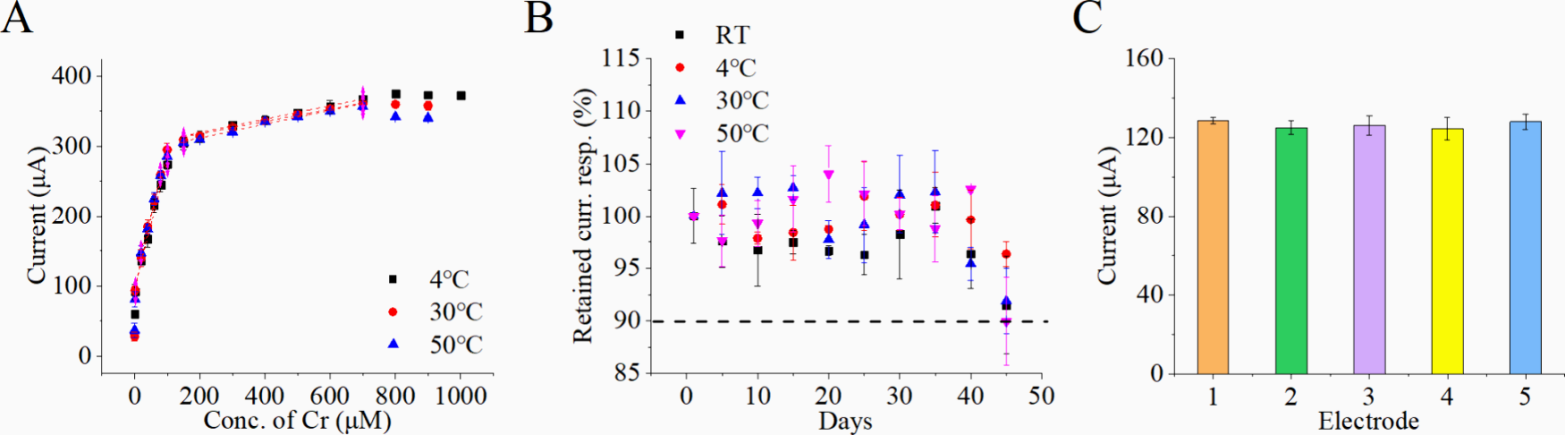
(A) Calibration curve of creatinine sensors modified with styrenyl-triphenylphosphonium-based ionic liquid (STPP-IL) and Nafion, evaluated at 4, 30, and 50 °C. (B) Peak current variation during cyclic voltammetry at room temperature for creatinine detection, measured for sensors stored under different conditions, including room temperature (RT), 4, 30, and 50 °C. (C) Reproducibility assessment of creatinine sensors by measuring 10 μM creatinine in artificial sweat using five independently fabricated electrodes. The data were obtained from three sensors with triplicate measurements. Error bars were calculated from the overall nine measurements.

The durability of creatinine sensors stored at different temperatures was evaluated over 45 days, as detailed in Tables S3–S6. Figure 5B illustrates the peak current variation during cyclic voltammetry at room temperature for creatinine detection under various storage conditions. The sensor retained 96.35%, 95.41%, 89.93%, and 91.46% of its initial current response after 45 days of storage at 4, 30, 50 °C, and room temperature, respectively. These results indicate superior stability, with only a slight decline in current response observed after approximately 40 to 45 days. Additionally, sensor reproducibility was assessed by measuring 10 μM creatinine in artificial sweat using five independently prepared sensors. The relative error was 1.3%, demonstrating excellent consistency (Figure 5C). The sensor’s robustness against temperature fluctuations supports its practical applicability, allowing storage between 4 and 50 °C without stringent temperature control. Furthermore, this sensor exhibits a longer operational lifetime compared to previously reported devices (Table S1). The sensor’s stability across a range of temperatures was attributed to the inherent robustness of its components, including CuONP, STPP-IL, and Nafion. Covalent immobilization of STPP-IL prevented material loss, ensuring consistent performance for point-of-care testing. The excellent thermal robustness is essential for wearable devices that are often exposed to temperature fluctuations due to body heat, environmental conditions, or storage scenarios. Our results suggest that the proposed sensor can maintain reliability without requiring strict cold storage or temperature control, thereby enhancing its practicality for decentralized or on-body applications. The ability to tolerate elevated temperatures such as 50 °C further supports its potential deployment in warm climates or during physical activity, where surface temperatures may rise significantly. Overall, the minimal variation in performance across a broad temperature range underscores the sensor’s feasibility for long-term, real-world use in wearable health monitoring platforms.

The ability in detecting low concentration creatinine and the long-term stability in various temperature conditions demonstrate the excellent practical applicability of our sensor. Commercial creatinine colorimetric kits from Thermo Fisher Scientific, Enzo Life Sciences, and Bio-Techne, which exhibited detection ranges of 44 to 354 μM, 27.4 to 1769 μM, and 27.6 to 1769 μM, respectively, are not suitable for detecting sweat creatinine. Additionally, the commercial kits required cold storage. In contrast, our proposed sensor operated reliably across a wider temperature range without the need for strict storage conditions. Its extended operational stability, reproducibility, and broad detection range supported its potential for noninvasive, wearable monitoring and one-step clinical assessment of creatinine under both physiological and pathological conditions.

## Conclusions

This study represents a significant advancement in nonenzymatic creatinine sensing by utilizing copper­(II) oxide (CuO) as a catalyst and integrating styrenyl-triphenylphosphonium-based ionic liquid (STPP-IL) and Nafion to enhance selectivity. Notably, this is the first study to combine ionic liquids with electrografting in electrochemistry to create a selective layer, significantly improving sensor performance.

The STPP-IL was synthesized and characterized using NMR spectroscopy, while SEM and EDS analyses confirmed its uniform distribution on the electrode surface. Selectivity assessments demonstrated that STPP-IL and Nafion effectively eliminated interference from common biomolecules, overcoming the limitations of existing nonenzymatic sensors. After optimization, the sensor achieved a low detection error of 3.69% in the presence of all interferents. The modified sensor exhibited excellent sensitivity, with values of 1.81 μM/μA (1.5–100 μM) and 0.09 μM/μA (150–800 μM) for creatinine detection. Recovery rates ranging from 97% to 105% in artificial sweat samples further validated its accuracy. Most importantly, the sensor was successfully used to quantify creatinine in natural sweat samples, confirming its potential for real-world applications. Long-term stability tests demonstrated that the sensor retained over 90% of its initial current response after 40 days, ensuring extended usability. Additionally, reproducibility experiments showed a relative error of only 1.3% across five independent sensors, confirming high reliability and consistency.

These findings highlight the enhanced selectivity, stability, and reproducibility of the modified sensor, making it a promising tool for reliable creatinine detection. This research contributes to the advancement of electrochemical biosensing technologies and has broader implications for clinical diagnostics, particularly in kidney function monitoring, ultimately improving patient outcomes.

## Supplementary Material



## Notes

Figure S1: ^13^C NMR spectrum of styrenyl-triphenylphosphonium chloride ([STPP]­Cl); Figure S2: comparison of creatinine detection performance using CuO electrodes, CuO electrodes modified with STPP-IL, and CuO electrodes modified with both STPP-IL and Nafion; Table S1: comparison of the proposed Nafion/STPP-IL/CuO/RGO sensor with existing creatinine sensors; Table S2: recovery rates of 10 μM creatinine in artificial sweat samples containing known concentrations of interferents; Tables S3–S6: durability assessment of creatinine sensors stored at 4, 30, and 50 °C, and under ambient conditions, based on peak current variations in cyclic voltammetry over 40 to 55 days at room temperature (PDF)
