# Supplementary material for: High-Selectivity Nonenzymatic Creatinine Sensor Using Electrografted Ionic Liquid and Nafion for Reliable Clinical Diagnostics
Source: ACS Sens. 2025 Aug 5;10(8):6020–8. doi: 10.1021/acssensors.5c01503 (PMC12379168; doi:10.1021/acssensors.5c01503)
Supplement: Supplementary file 1 [file se5c01503_si_001.pdf]

Supporting Information

High-Selectivity Non-Enzymatic Creatinine Sensor Using Electrografted Ionic Liquid and Nafion for Reliable Clinical Diagnostics.

Shih-Hao Lin[a], Jing-Chun Wang[a], Zong-Hong Lin[c], Fu-Cheng Kao[d,e] and Hsiang-Yu Wang[a,b*]

[a]Department of Engineering and System Science, National Tsing Hua University, 101, Section 2, Kuang-Fu Road, Hsinchu 300044, Taiwan

[b]Institute of Nuclear Engineering, National Tsing Hua University, 101, Section 2, Kuang-Fu Road, Hsinchu 300044, Taiwan

[c]Department of Biomedical Engineering, National Taiwan University, No.1, Section 4, Roosevelt Road, Taipei 106319, Taiwan

[d]Department of Orthopaedic Surgery, Chang Gung Memorial Hospital, No. 5, Fuxing St., Guishan Dist., Taoyuan City 33305, Taiwan

[e]College of Medicine, Chang Gung University, No.259, Wenhua 1st Rd., Guishan Dist., Taoyuan City 33302, Taiwan

[*]Email address: hywang@ess.nthu.edu.tw

Table of Content

Figure S1. The ^{13}C NMR spectrum of styrenyl-triphenylphosphonium chloride ([STPP]Cl).

Figure S2. Comparison of the performance of creatinine detection with the CuO electrodes, CuO electrodes modified with STPP-IL, and CuO electrodes modified with both STPP-IL and Nafion.

Table S1. Comparisons of the proposed Nafion/STPP-IL/CuO/RGO sensor with existing creatinine sensors.

Table S2. Recovery rates of 10 µM creatinine in artificial sweat samples containing known concentrations of interferents.

Table S3. Durability of creatinine sensors stored at 4°C, assessed by peak current during cyclic voltammetry at room temperature over 55 days.

Table S4. Durability of creatinine sensors stored at 30°C, assessed by peak current during cyclic voltammetry at room temperature over 50 days.

Table S5. Durability of creatinine sensors stored at 50°C, assessed by peak current during cyclic voltammetry at room temperature over 45 days.

Table S6. Durability of creatinine sensors stored at ambient environment, assessed by peak current during cyclic voltammetry at room temperature over 40 days.

Reference

The ^{13}C spectra of the synthesized ionic liquid were acquired using an AVANCE-500 NMR spectrometer (Bruker Inc., Germany). Approximately 10 mg of the ionic liquid was dissolved in deuterated dimethyl sulfoxide (d-DMSO) and transferred to a 5 mm NMR tube for analysis. Chemical shifts were reported relative to d-DMSO (39.5 ppm for ^{13}C NMR), with 2034 scans for ^{13}C NMR.

The molecular structure of styrenyl-triphenylphosphonium chloride ([STPP]Cl) was confirmed via ^{13}C NMR analysis (Figure S1). Peaks at 135.0 and 131.9 ppm were attributed to carbon atoms in the benzene ring of the triphenylphosphine moiety, while the peak at 119.5 ppm corresponded to the carbon atoms adjacent to the phosphorus ion near the chloride. A peak at 130.2 ppm was assigned to carbon atoms in the benzene ring of the methyl styrene structure, and the peak at 114.3 ppm indicated the presence of a sp^2 carbon in the alkene, representing the structure's terminus. The peak observed at 30.8 ppm is attributed to the carbon atom in methyl styrene adjacent to the chloride. Following the quaternization reaction, a significant shift in the peak corresponding to this carbon atom was noted, indicating a decrease in affinity due to the reduced electronegativity of the phosphorus ion. Compared to the signal from the similarly positioned carbon atom at 46.2 ppm in 4-chloromethyl styrene, this shift further substantiates the successful synthesis of STPP-IL (4-Vinylbenzyl chloride).

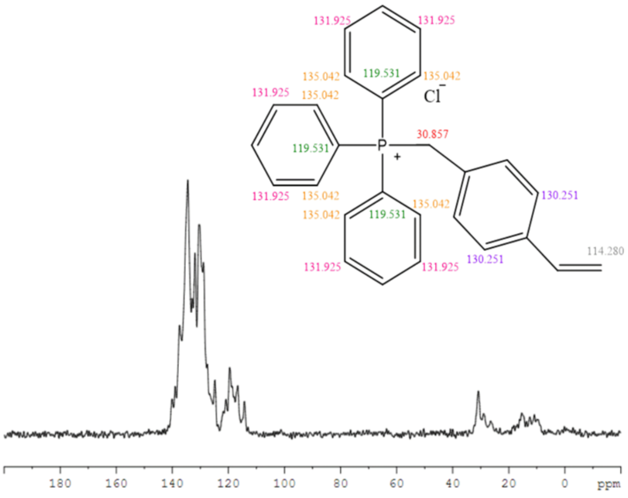

Figure S1. The ^{13}C NMR spectrum of styrenyl-triphenylphosphonium chloride ([STPP]Cl), including the structural formula with labeled carbon atoms corresponding to the signals observed in the spectrum. $^{13}C\{^{1}H\}$ NMR (d-DMSO, 500 MHz): δ 26.5, 29.0, 30.8, 114.3, 116.7, 117.9, 118.6, 119.5, 120.9, 121.8, 124.8, 126.6, 127.5, 128.5, 129.5, 130.2, 130.4, 131.9, 132.8, 134.4, 135.0, 137.4, 138.9, 140.1 ppm.

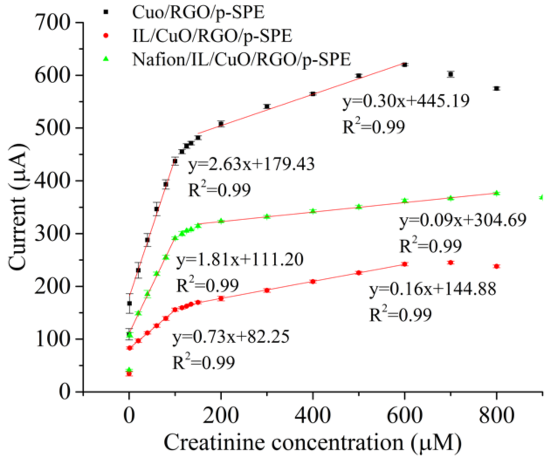

(a)

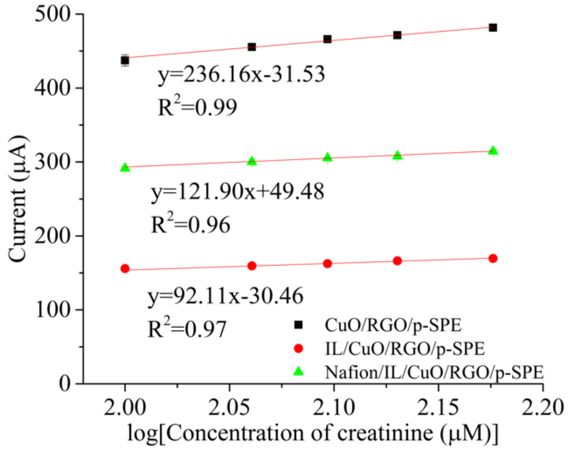

(b)

Figure S2. (a) Comparison of the calibration curves for the CuO electrode, CuO electrode modified with STPP-IL, and CuO electrode modified with both STPP-IL and Nafion. (b) The blowup of the calibration curve and logarithmic transformation of the concentration data in (a) between 100 and 150 μM.

Table S1. Comparisons of the proposed Nafion/STPP-IL/CuO/RGO sensor with existing creatinine sensors.

**Catalyst / Electrode**	**Linear range (μM)**	**Sensitivity (μA / μM*cm^2)**	**R^2**	**Interfering Agents**	**Operational stability(days)**	**Reference**
CuO/IL/RGO	500 ~ 5000 & 7000 ~ 30000	2.25×10^{-4} & 7.11×10^{-5}	0.9999 & 0.9997	Urine	35	[1]
PAA/Nafion/Cu	1 ~ 2000	0.481	0.997	Artificial Saliva	30	[2]
PVA/PEDOT/PSS/Cu	0.4 ~ 960	0.6428	0.996	Artificial Sweat	30	[3]
(CDI)/PANI/Nafion/Cu	1 ~ 125	85000	0.997	Serum	3	[4]
(CA-CI-SO)/PVA/PEG	66 ~ 810	9.83×10^{-4}	0.9802	Serum	33	[5]
CuO/PVP/MAA	0.5 ~ 200	0.176	0.997	Urine	14	[6]
Fe_3O_4/PANI	0.02 ~ 1	882.65	0.996	Human plasma	15	[7]
$[Fe(CN)_6]^{3-/4-}$/RGO	560 ~ 50000 & 230 ~ 50000	4.83 & 0.118 (μC / μM)	NA	Urine	NA	[8]
Nafion/STPP-IL/CuO/RGO	1.5 ~ 100 & 150 ~ 800	45.25 & 2.25	0.9998 & 0.9961	Artificial Sweat	40	This study

RGO = reduced graphene oxide, PAA = Poly(acrylic acid), PVA = Polyvinyl alcohol, PEDOT = Poly(3,4-ethylenedioxythiophene), PSS = polystyrene sulfonate, CDI = creatinine deiminase, PANI = Polyaniline, CA = creatinine amidohydrolase, CI = creatine amidohydrolase, SO = sarcosine oxidase, PEG = polyethylene glycol, PVP = polyvinylpyrrolidone, MAA = methacrylic acid, $[Fe(CN)_6]^{3-}$ = ferro-/ferricyanide.

Table S2. Recovery rates of 10 μM creatinine in artificial sweat samples containing known concentrations of interferents (0.09 mM glucose, 50 μM uric acid, 22.2 mM urea, 5 mM lactic acid, and 10 μM ascorbic acid) using creatinine sensors electro-decorated with STPP-IL and Nafion.

Sample label	Added concentration of creatinine (μM)	Average peak current (μA)	Estimated concentration of creatinine (μM)	Recovery (%)	RSD (%)
Sample 1	9.4	118.37 ± 1.11	9.12 ± 0.62	97.02	0.94
Sample 2	12	123.90 ± 1.13	12.19 ± 0.63	102.25	1.06
Sample 3	14	128.37 ± 1.98	14.68 ± 1.53	104.86	2.14
Sample 4	16	131.93 ± 0.97	16.66 ± 0.54	104.13	0.73
Sample 5	18	135.83 ± 0.52	18.82 ± 0.29	104.56	0.39

RSD = Relative standard deviation

Table S3. Durability of creatinine sensors stored at 4°C, assessed by peak current during cyclic voltammetry at room temperature over 55 days.

Days	Current (μA)	Retained current response(%)	RSD(%)
1	128.70 ± 3.69	100.00	0.03
5	127.80 ± 2.41	99.30	1.87
10	123.63 ± 0.74	96.06	0.58
15	124.33 ± 3.31	96.61	2.57
20	126.13 ± 0.45	98.70	0.35
25	126.00 ± 4.14	101.91	3.22
30	124.56 ± 2.05	100.19	1.60
35	130.06 ± 4.01	101.06	3.11
40	128.23 ± 3.65	99.64	2.84
45	124.00 ± 1.53	96.35	1.19
50	119.4 ± 4.94	92.77	3.84
55	103.33 ± 1.56	80.29	1.21

RSD = Relative standard deviation

Table S4. Durability of creatinine sensors stored at 30°C, assessed by peak current during cyclic voltammetry at room temperature over 50 days.

Days	Current (μA)	Retained current response(%)	RSD(%)
1	123.56 ± 4.07	100.00	3.29
5	126.30 ± 4.95	102.21	4.01
10	126.33 ± 1.86	102.24	1.47
15	126.93 ± 1.42	102.72	1.12
20	123.43 ± 2.29	99.89	1.80
25	125.23 ± 4.41	101.35	3.57
30	126.10 ± 4.66	102.05	3.72
35	126.40 ± 4.88	102.29	3.95
40	117.90 ± 1.93	95.41	1.56
45	113.53 ± 3.80	91.88	3.07
50	92.42 ± 1.92	74.80	1.55

RSD = Relative standard deviation

Table S5. Durability of creatinine sensors stored at 50°C, assessed by peak current during cyclic voltammetry at room temperature over 45 days.

Days	Current (μA)	Retained current response(%)	RSD(%)
1	123.76 ± 3.62	100.00	2.93
5	120.83 ± 3.05	97.63	2.46
10	122.86 ± 2.66	99.27	2.20
15	125.76 ± 3.88	101.62	3.16
20	125.70 ± 3.40	104.03	2.70
25	125.50 ± 3.89	102.14	3.10
30	126.03 ± 1.89	100.21	1.51
35	122.23 ± 3.94	98.76	3.19
40	126.96 ± 0.39	102.59	0.31
45	111.30 ± 5.17	89.93	4.18

RSD = Relative standard deviation

Table S6. Durability of creatinine sensors stored at ambient environment, assessed by peak current during cyclic voltammetry at room temperature over 45 days.

Days	Current (μA)	Retained current response(%)	RSD(%)
1	127.66 ± 2.62	100.00	2.62
5	124.56 ± 3.16	97.57	2.48
10	123.50 ± 4.42	96.74	3.46
15	124.46 ± 1.43	97.49	1.12
20	123.40 ± 0.57	96.66	0.44
25	122.93 ± 2.43	96.29	1.90
30	125.43 ± 5.41	98.25	4.24
35	128.93 ± 2.23	100.99	1.75
40	123.03 ± 4.20	96.37	3.29
45	116.76 ± 5.93	91.46	4.65

SD = standard deviation of three replicate value.

Reference

(1) Teekayupak, K.; Aumnate, C.; Lomae, A.; Preechakasedkit, P.; Henry, C. S.; Chailapakul, O.; Ruecha, N. Portable Smartphone Integrated 3D-Printed Electrochemical Sensor for Nonenzymatic Determination of Creatinine in Human Urine. *Talanta* 2023, *254*, 124131.

(2) Kalasin, S.; Sangnuang, P.; Khownarumit, P.; Ming Tang, I.; Surareungchai, W. Salivary Creatinine Detection Using a Cu(I)/Cu(II) Catalyst Layer of a Supercapacitive Hybrid Sensor: A Wireless IoT Device To Monitor Kidney Diseases for Remote Medical Mobility. *ACS Biomaterials Science & Engineering* 2020. https://doi.org/10.1021/acsbiomaterials.0c00864.

(3) *[No title]*. https://doi.org/10.1021/acsbiomaterials.0c01459 (accessed 2024-10-06).

(4) Creatinine and Urea Biosensors Based on a Novel Ammonium Ion-Selective Copper-Polyaniline Nano-Composite. *Biosensors and Bioelectronics* 2016, *77*, 505–511.

(5) Uric Acid and Creatinine Biosensors with Enhanced Room-Temperature Storage Stability by a Multilayer Enzyme Matrix. *Anal. Chim. Acta* 2022, *1227*, 340264.

(6) Nontawong, N.; Amatatongchai, M.; Thimoonnee, S.; Laosing, S.; Jarujamrus, P.; Karuwan, C.; Chairam, S. Novel Amperometric Flow-Injection Analysis of Creatinine Using a Molecularly-Imprinted Polymer Coated Copper Oxide Nanoparticle-Modified Carbon-Paste-Electrode. *J. Pharm. Biomed. Anal.* 2019, *175*, 112770.

(7) Novel Electrochemical Sensing Platform Based on Magnetic Field-Induced Self-Assembly of Fe3O4@Polyaniline Nanoparticles for Clinical Detection of Creatinine. *Biosensors and Bioelectronics* 2014, *56*, 180–185.

(8) Bezinge, L.; Tappauf, N.; Richards, D. A.; Shih, C.-J.; deMello, A. J. Rapid Electrochemical Flow Analysis of Urinary Creatinine on Paper: Unleashing the Potential of Two-Electrode Detection. *ACS Sens.* **2023**, *8* (10), 3964–3972.
